# Reducing anxiety and improving self-acceptance in children and adolescents with osteosarcoma through group drawing art therapy

**DOI:** 10.3389/fpsyg.2023.1166419

**Published:** 2023-04-17

**Authors:** Xin Liu, Lihong Sun, Xinhui Du, Chang Zhang, Yijia Zhang, Xiaoxia Xu

**Affiliations:** ^1^Department of Bone and Soft Tissue, The Affiliated Cancer Hospital of Zhengzhou University, Henan Cancer Hospital, Zhengzhou, Henan, China; ^2^Zhengzhou Municipal Hospital of Pains on Neck-Shoulder-Waist, Zhengzhou, Henan, China; ^3^Department of Integrated Chinese and Western Medicine, The Affiliated Cancer Hospital of Zhengzhou University, Henan Cancer Hospital, Zhengzhou, Henan, China; ^4^Department of Nursing, The Affiliated Cancer Hospital of Zhengzhou University, Henan Cancer Hospital, Zhengzhou, Henan, China

**Keywords:** drawing, art therapy, osteosarcoma, anxiety, self-acceptance

## Abstract

**Purpose:**

This study explored the effect of group drawing art therapy (GDAT) on anxiety and self-acceptance in children and adolescents with osteosarcoma.

**Methods:**

Using a randomized experimental study design, 40 children and adolescents with osteosarcoma who were treated in our hospital from December 2021 to December 2022 were selected as the research objects, including 20 in the intervention group and 20 in the control group. The control group received routine care for osteosarcoma, while the intervention group participated in eight sessions of GDAT, twice a week, 90–100 min each, in addition to routine care for osteosarcoma. A screening for children’s anxiety disorders (SCARED) and a self-acceptance questionnaire (SAQ) were used to evaluate the patients before and after the intervention.

**Results:**

After 8 weeks of GDAT, the SCARED total score in the intervention group was 11.30 ± 8.603, and that in the control group was 22.10 ± 11.534. The difference between the two groups was statistically significant (t = -3.357, *P* < 0.05). In the intervention group, the SAQ total score was 48.25 ± 4.204, with self-acceptance and self-evaluation factor scores of 24.40 ± 2.521 and 23.85 ± 2.434, respectively. In the control group, the SAQ total score was 42.20 ± 4.047; the self-acceptance factor score was 21.20 ± 3.350 and that of the self-evaluation factor was 21.00 ± 2.224. The differences between the two groups were statistically significant (t = 4.637, *P* < 0.001; t = 3.413, *P* < 0.05; t = 3.866, *P* < 0.001, respectively).

**Conclusion:**

Group drawing art therapy can reduce anxiety and improve the levels of self-acceptance and self-evaluation in children and adolescents with osteosarcoma.

## 1. Introduction

Childhood and adolescence are important stages of physical and mental development and are prone to psychological and behavioral problems such as anxiety, low self-acceptance, low self-esteem, and social withdrawal (Lazor et al., 2021; Forrest et al., 2022). Anxiety disorders are characterized by symptoms of anxiety, fear, nervousness, and worry, as well as physical symptoms such as palpitations, shortness of breath, dizziness, and muscle tension (Birmaher et al., 1997; Lewandowska et al., 2021). Self-acceptance means that an individual can objectively accept and view himself, establish self-value based on self-recognition, respect and appreciate himself (Huang et al., 2021; Kelemen and Shamri-Zeevi, 2022). People with low self-acceptance develop psychological and behavioral problems such as low self-esteem and social withdrawal. Studies have shown that children and adolescents with cancer more likely to experience these psychological problems than those without cancer (Lewandowska et al., 2021; Raybin et al., 2022). Osteosarcoma is a severe teratogenic and fatal disease that occurs mainly in children and adolescents (Tian et al., 2020). Compared to patients with other non-teratogenic cancers, children and adolescents with osteosarcoma are bound to suffer greater psychological stress and may be more prone to psychological abnormalities (Lewandowska et al., 2021; Wu et al., 2022).

By providing information, support, and encouragement, healthcare providers and psychotherapists can help these children and adolescents cope with the rapidly changing psychological issues and challenges, thereby improving the effectiveness of cancer treatment and quality of life (Bar-Sela et al., 2007; Raybin et al., 2023). Interventions for this special type of patients should be professional, diverse, and in line with the developmental characteristics of children’s psychology (de Witte et al., 2021; Bekar et al., 2022; Cheng et al., 2022). Drawing art therapy refers to a non-linguistic psychotherapy technique that reflects an individual’s ability, personality, interests, concerns, and conflicts through painting, creative artistic activities, and their feedback on painting works. It can be used to improve the person’s cognitive level, cultivate self-esteem and self-awareness, augment emotional resilience, promote insight, enhance social skills, and reduce emotional conflict and psychological pain (Jiang et al., 2020; Zhang et al., 2022). Compared with traditional psychotherapy, drawing art therapy is not limited by language, age, place environment, cognitive ability, and disease. Furthermore, it is easily accepted by patients, with minimal resistance and simple treatment implementation, and is gradually being used in clinical treatment (Gürcan and Atay Turan, 2021; Raybin et al., 2022). Research has established that interventions using drawing art therapy have obvious effects in some individuals (Bar-Sela et al., 2007; Zhang et al., 2022).

Currently, there are no intervention studies on drawing psychotherapy for children and adolescents with osteosarcoma, which may be related to the low incidence of the disease. Consequently, this study aimed to intervene and guide children and adolescents with osteosarcoma by using group drawing art therapy (GDAT) for their low self-esteem, anxiety, and other emotions, to help them relieve their emotional and psychological problems during treatment.

## 2. Materials and methods

### 2.1. Participants

**FIGURE 1:**
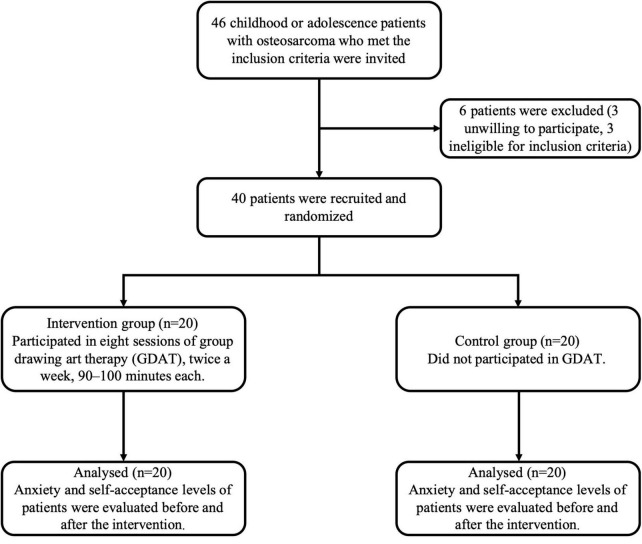
The consort diagram of the participants of this study.

This is a randomized, controlled, non-blind clinical study. The consort diagram of this study is shown as Figure 1. In this study, child and adolescent patients with osteosarcoma who met the inclusion criteria were recruited from December 2021 to December 2022. Consequently, patients (1) aged 7–18, (2) diagnosed with osteosarcoma through pathological classification, and (3) who had undergone chemotherapy and surgery were included in the study. The exclusion criteria were as follows: (1) severe intellectual disability; (2) critical stage, myelosuppression stage, or with serious complications; and (3) past psychotherapy experience.

After being fully informed of the contents and procedures of GDAT and routine care, patients and their respective guardians signed an informed consent form. This study was approved by the Ethics Committee of Henan Cancer Hospital (approval number: 2021-KY-0176-003).

### 2.2. Procedure

The patients were randomly divided into control and intervention groups. Random assignment was carried out by an investigator who was blinded to the patient’s information. The study was conducted in a non-blinded condition, since the used instruments were self-report and children could not be blinded due to the nature of the painting therapy. The control group received routine nursing of osteosarcoma, while the intervention group received GDAT (Figure 1).

This study conducted GDAT on groups of 4–7 patients twice a week for 60–90 min. Each therapy session was divided into three stages: (1) a warm-up activity stage, which took approximately 20 min. In this stage, the psychotherapist led the patient to relax by playing ice-breaking games. (2) The theme drawing stage, of about 30–40 min, in which the drawing therapist introduced the scheduled drawing theme to the patients, and the patients drew freely. The content of the drawing was decided by the patient, without the therapist guiding it; the latter merely accompanied the patient to observe the order and content of the drawing. The intervention group received eight GDAT sessions; the themes are detailed in Table 1. The purpose was to guide patients to release various complex emotions generated during hospitalization through a creative process. (3) The ending stage took approximately 20 min. In this stage, the patients were guided to share their work, experience, feelings, and outcome of the drawing therapy, to relieve anxiety and reshape hope.

**TABLE 1:** List of themes for group drawing art therapy.

Sequence	Themes	Purpose
1	Who am I?	Guide patients to recognize and accept themselves, so that they can share their drawings and gain understanding and support from others.
2	The emotion hidden in my heart	Guide the patients to make friends with emotion, help them transform the squeezed emotion, learn to accept it, and alleviate the pain caused by the current disease.
3	If I were a tree	Guide patients to improve their endurance and energy by imagining themselves as a tree.
4	Superhero	Guide patients to imagine their own god of protection and enhance their sense of security and belonging.
5	The one who loves me most	Encourage family members to participate, guide patients to transform their inner expectations into realistic images, to feel and transmit love.
6	What I want to do most	Guide patients to accept the imperfections in life and cherish the present through sharing life regrets and expectations.
7	Making friends with disease	Guide patients to face and understand the disease, overcome their fear and resistance to it, and enhance their confidence in overcoming the disease.
8	Future me	Give support and encouragement to make children full of hope for the future.

### 2.3. Evaluation

The patients’ age, age at diagnosis, weight, sex, family status, disease, and other basic information were recorded.

Patient anxiety was assessed using the Screen for Child Anxiety Related Emotional Disorders (SCARED) scale (Birmaher et al., 1997). The scale’s 41 items were divided into five factors: somatic panic, generalized anxiety, dissociative anxiety, social phobia, and school phobia. Each factor is scored at Level 3, with the options “do not have this problem,” “sometimes,” and “often” receiving 0, 1, and 2 points, respectively. The score for each item was added to obtain the total score of the scale. The higher the total score of the scale, the higher is the level of individual anxiety.

Patient self-acceptance was assessed using a self-acceptance questionnaire (SAQ; Cong and Gao, 1999; Chen et al., 2017). The SAQ was used to evaluate an individual’s acceptance level of their current state. The scale consists of 16 items, which can be divided into two factors: self-acceptance and self-evaluation. The scale adopts a Likert 4-level scoring method, with each item receiving from 1 (*very same*) to 4 (*very opposite*) points. The total score of the scale ranged from 16 to 64 points; the higher the score, the higher the level of self-acceptance.

The SCARED and SAQ scale scores were collected by two specially trained researchers before the first GDAT session and on the day after the end of the eighth GDAT session.

### 2.4. Statistical methods

This study employed the data collation software EXCEL (Microsoft, Redmond, WA, United States), which ensured the accuracy of the data through double entry. SPSS 21.0 (IBM, Amenk, NY, United States) was used for data analysis. The measurement data conformed to the normal distribution; the mean ± standard deviation was used for statistical description, while the frequency and constituent ratio of the counting data were used for statistical description. The measurement data were consistent with a normal distribution and homogeneity of variance. A paired *t*-test was used to compare intra-group differences, and a *t*-test of two independent samples was used to compare inter-group differences. The chi-square test was used to compare differences between the groups. The inspection level was considered α = 0.05.

## 3. Results

### 3.1. The basic characteristics of the groups

Forty patients aged 7–18 were included in this study. There were 20 boys and 20 girls, with an average age of 12.10 years. All the enrolled patients underwent chemotherapy or surgery combined with chemotherapy. There was no significant difference in the general data between the control group (20 patients) and the intervention group (20 patients; *P* > 0.05; Table 2).

**TABLE 2:** The basic characteristics of the intervention and control groups.

Characteristics	Intervention group (*n* = 20)	Control group (*n* = 20)	*P*
**Gender (%)**			
Male	9 (45.0)	11 (55.0)	0.527
Female	11 (55.0)	9 (45.0)	
Age (years, mean ± standard deviation)	11.40 ± 3.331	12.95 ± 2.762	0.117
Age at diagnosis (years, mean ± standard deviation)	10.70 ± 3.404	12.40 ± 2.542	0.082
Weight (kg)	45.750 ± 16.708	48.300 ± 12.791	0.591
**Family monthly earning (¥, %)**			
≤5,000	6 (30.0)	12 (60.0)	0.162
5,000–10,000	7 (35.0)	4 (20.0)	
>10,000	7 (35.0)	4 (20.0)	
**Medical payment methods (%)**			
Self-paying	1 (5.0)	3 (15.0)	0.139
Medical insurance	19 (95.0)	17 (85.0)	
**Other family members with cancer (%)**			
Yes	0 (0.0)	1 (5.0)	0.311
No	20 (100.0)	19 (95.0)	
**Site of the osteosarcoma (%)**			
Femur	10 (50.0)	7 (35.0)	0.277
Tibia	7 (35.0)	5 (25.0)	
Humerus	0 (0.0)	2 (10.0)	
Other bone	3 (15.0)	6 (30.0)	
**Treatment (%)**			
Surgery plus chemotherapy (%)	16 (80.0)	15 (75.0)	0.972
Chemotherapy	2 (10.0)	3 (15.0)	
Surgery	1 (5.0)	1 (5.0)	
Other	1 (5.0)	1 (5.0)	
**Presence of metastasis (%)**			
Yes	9 (45.0)	10 (50.0)	0.752
No	11 (55.0)	10 (50.0)	

### 3.2. SCARED scale scores comparison between the two groups before and after intervention

#### 3.2.1. Intra-group comparison

The total scores of the SCARED (*P* < 0.001), somatization/panic factor (*P* < 0.001), generalized anxiety factor (*P* < 0.05), dissociative anxiety factor (*P* < 0.05), and social terror factor (*P* < 0.001) in the intervention group were significantly lower than those before the intervention (Table 3). The total scores of the SCARED, somatization/panic, generalized anxiety, dissociative anxiety, social phobia, and school phobia factors in the control group after the intervention were not significantly different from those before the intervention (*P* > 0.05).

**TABLE 3:** Screening for children’s anxiety disorders (SCARED) scale score comparison between the intervention and control groups.

	Total score of SCARED		*P*	Somatization/Panic factor		*P*
	**Before intervention**	**After intervention**		**Before intervention**	**After intervention**	
Intervention group	24.75 ± 17.103	11.30 ± 8.603	< 0.001	6.05 ± 5.871	2.35 ± 2.889	< 0.001
Control group	20.05 ± 9.833	22.10 ± 11.534	0.432	5.15 ± 4.804	5.25 ± 3.683	0.907
*P*	0.293	<0.05		0.599	<0.05	
	**Generalized anxiety factor**		***P***	**Dissociative anxiety factor**		***P***
	**Before intervention**	**After intervention**		**Before intervention**	**After intervention**	
Intervention group	4.85 ± 3.815	2.25 ± 2.314	<0.05	5.15 ± 3.774	2.70 ± 2.250	<0.05
Control group	4.35 ± 3.014	4.25 ± 2.552	0.871	3.90 ± 2.426	4.30 ± 2.975	0.504
*P*	0.648	<0.05		0.220	0.063	
	**Social phobia factor**		***P***	**School terror factor**		***P***
	**Before intervention**	**After intervention**		**Before intervention**	**After intervention**	
Intervention group	7.30 ± 3.246	3.45 ± 2.762	<0.001	1.40 ± 1.930	0.55 ± 0.887	0.063
Control group	5.15 ± 2.390	7.00 ± 5.858	0.129	1.50 ± 1.539	1.30 ± 1.418	0.507
*P*	<0.05	<0.05		0.857	0.052	

Mean ± standard deviation was used for the statistical analysis. SCARED, screen for child anxiety-related emotional disorders.

#### 3.2.2. Comparison between groups

Before intervention, there was no significant difference in the total scores of the SCARED, the somatization/panic, generalized anxiety, dissociative anxiety, and school terror factors between the two groups (*P* > 0.05). Before the intervention, the social phobia score in the intervention group was significantly higher than that in the control group (*P* < 0.05). After the intervention, the total scores on the SCARED (*P* < 0.05), somatization/panic (*P* < 0.05), generalized anxiety (*P* < 0.05), and social phobia (*P* < 0.05) factors in the intervention group were significantly lower than those in the control group. Although the scores of the dissociative anxiety and school terror factors in the intervention group were lower than those in the control group, the difference was not statistically significant (*P* > 0.05; Table 3).

### 3.3. SAQ score comparison between the two groups before and after intervention

#### 3.3.1. Intra-group comparison

After the intervention, the SAQ total score (*P* < 0.001) and self-acceptance factor score (*P* < 0.001) in the intervention group were significantly higher than those before the intervention. Although the self-evaluation factor score in the intervention group after the intervention was higher than that before, the difference was not statistically significant (*P* > 0.005). The SAQ total score, and the self-acceptance and self-evaluation factor scores in the control group after the intervention were not significantly different from those before the intervention (*P* > 0.05; Table 4).

**TABLE 4:** Self-acceptance questionnaire (SAQ) score comparison between the intervention and control groups.

	SAQ total score		*P*	Self-acceptance factor		*P*	Self-evaluation factor		*P*
	**Before intervention**	**After intervention**		**Before intervention**	**After intervention**		**Before intervention**	**After intervention**	
Intervention group	42.95 ± 6.203	48.25 ± 4.204	<0.001	20.15 ± 3.438	24.40 ± 2.521	<0.001	22.80 ± 3.806	23.85 ± 2.434	0.153
Control group	42.65 ± 4.870	42.20 ± 4.047	0.579	20.90 ± 3.851	21.20 ± 3.350	0.734	21.75 ± 3.919	21.00 ± 2.224	0.279
*P*	0.866	<0.001		0.520	<0.05		0.395	<0.001	

Mean ± standard deviation was used for the statistical analysis. SAQ, self-acceptance questionnaire.

#### 3.3.2. Comparison between groups

Before the intervention, there were no significant differences between the two groups in the total SAQ, self-acceptance, and self-evaluation scores (*P* > 0.05). After the intervention, the total scores of the SAQ scale (*P* < 0.001), self-acceptance factor (*P* < 0.05), and self-evaluation factor (*P* < 0.001) in the intervention group were significantly higher than those in the control group.

## 4. Discussion

This study found that after eight GDAT sessions, the total scores of the anxiety scale, and the scores for somatization/panic, generalized anxiety, and social phobia factors of children and adolescents with osteosarcoma in the intervention group were significantly lower than those in the control group. The total scores of the self-acceptance scale, and those on the self-acceptance and self-evaluation factors of children and adolescents with osteosarcoma in the intervention group were significantly higher than those in the control group.

Children and adolescent patients with osteosarcoma are more prone to anxiety due to their high degree of disease malignancy, rapid growth, early metastasis, poor prognosis, limb deformity, and pain (Lewandowska et al., 2021; Wu et al., 2022). Drawing therapy can use non-verbal visual arts to deeply explore individual emotions, touch patients’ inner subconscious, and correct unpleasant emotional experiences. It allows patients to develop symbolic language and learn new ways of expressing emotions (Bar-Sela et al., 2007; Bozcuk et al., 2017). GDAT enables patients to express various negative emotions that arise from being hospitalized and treated for illness—fear of illness, pain from illness and treatment, and anxiety about the uncertainty of physical recovery—through colors, lines, images, and stories. Consequently, the visible and invisible anxiety become a tangible and telling picture, and finally, the emotion itself weakens or disappears (Raybin et al., 2022; Zhang et al., 2022). Several studies have shown that different forms of drawing interventions can effectively reduce anxiety and depressive symptoms in adolescent cancer patients (Bozcuk et al., 2017; Forouzandeh et al., 2020; Gürcan and Atay Turan, 2021). The results of this study are consistent with those of previous research; that is, after eight sessions of group drawing therapy, the anxiety scale score of the treated patient was significantly reduced. In this study, the themed activities of “Who am I?” and “The emotion hidden in my heart” were used to guide patients to understand themselves and their emotions, release various complex emotions during hospitalization, dissolve pain and confrontation, and help them weaken or eliminate negative repressed emotions.

Studies have shown that cancer patients generally have a low level of self-acceptance (Li et al., 2010). Self-acceptance means that an individual can objectively accept and view himself, establish self-value based on self-recognition, respect and appreciate himself (Huang et al., 2021; Kelemen and Shamri-Zeevi, 2022). Adolescent cancer patients are more likely to doubt their own worth and lower their level of self-acceptance (Lewandowska et al., 2021). The results of this study are consistent with previous findings; that is, providing psychological intervention support to cancer patients can significantly improve their level of self-acceptance (Bozcuk et al., 2017; Zhang et al., 2022). The process of drawing can correct discordant cognition through abstract image thinking and imagination to promote and improve the painter’s cognitive function and the degree of self-acceptance (Huang et al., 2021). This study guides patients to think about how to get along with the disease, stabilize their inner state, explore their personality traits, accept life imperfections, improve self-esteem, and realize self-appreciation and self-acceptance through drawing themed activities such as “What I want to do most,” “Make friends with the disease,” and “Future me.”

In addition to anxiety and self-acceptance, previous studies have shown that drawing therapy can also help reshape the hope of cancer patients, promote energy conversion, and guide patients to love life (Collette et al., 2021; Zhang et al., 2022; Raybin et al., 2023). This study uncovered similar findings, thus demonstrating increased levels of hope remodeling in patients who received GDAT. For example, a 9 years-old patient drew a closed road for the first time, thus indicating the rejection of himself and despair for the future. After four group drawing therapy sessions, the patient drew a scene of the school raising the national flag, thereby suggesting that the person had high hopes of returning to school.

The major limitations of this study are the limited sample size and the fact that it was conducted at a single institution. In future multi-center, large-sample randomized controlled trials, children can be classified more carefully, and more individualized themes can be designed according to the actual situation to better adjust for children’s inner anxiety. In addition, this study did not use objective research tools to assess the level of hope remodeling in patients. Future studies should further evaluate the impact of GDAT on hope remodeling and the sense of happiness in patients with sarcoma through objective and evaluable indicators.

## 5. Conclusion

Eight sessions of GDAT can significantly reduce the anxiety level of children and adolescents with osteosarcoma, while significantly improving their self-acceptance. It demonstrates that it is an effective intervention that can be widely used in the treatment and nursing of children and adolescents with osteosarcoma.

## Data availability statement

The original contributions presented in this study are included in the article/supplementary material, further inquiries can be directed to the corresponding author.

## Ethics statement

The studies involving human participants were reviewed and approved by Ethics Committee of Henan Cancer Hospital (approval number: 2021-KY-0176-003). Written informed consent to participate in this study was provided by the participants’ legal guardian/next of kin.

## Author contributions

XL and XX conceived and designed the study. XL, LS, CZ, and YZ collected, analyzed the data, and wrote the manuscript. XD and XX prepared the tables and revised the manuscript. All authors read and approved the submitted version.
